# Records and altitudinal assessment of *Amblyomma aureolatum* and *Amblyomma ovale* (Acari: Ixodidae) in the State of Rio de Janeiro, southeast Brazil

**DOI:** 10.1186/s13071-022-05250-6

**Published:** 2022-04-21

**Authors:** João L. H. Faccini, Hélio F. Santos, Lívio M. Costa-Junior, Socrates F. Costa-Neto, Wagner S. Tassinari, Hermes R. Luz

**Affiliations:** 1 Department of Animal Parasitology, Universidade Federal Rural do Rio de Janeiro (UFRRJ), Seropédica, RJ Brazil; 2 Postgraduate Program in Veterinary Sciences, UFRRJ, Seropédica, Brazil; 3 Northeast Biotechnology Network Postgraduate Program (RENORBIO), Federal University of Maranhão (UFMA), São Luís, MA Brazil; 4 Fundação Oswaldo Cruz (Fiocruz), Rio de Janeiro, RJ Brazil; 5 Department of Mathematics, UFRRJ, Seropédica, RJ Brazil; 6 Postgraduate Program in Health and Environment, UFMA, São Luís, MA Brazil; 7 Postgraduate Program in Biodiversity and Conservation, UFMA, São Luís, MA Brazil

**Keywords:** *Amblyomma aureolatum*, *Amblyomma ovale*, Altitude, Spotted fever, Atlantic forest

## Abstract

Information on the altitudinal distribution of the hard ticks *Amblyomma aureolatum* and *Amblyomma ovale* in Brazil is scarce and mainly limited to occasional records. In this study we report our evaluation of records on the altitudinal distribution of *A. aureolatum* and *A. ovale* collected from dogs and humans and directly from the environment (host-questing ticks), based on active or passive procedures. The collections were conducted in rural areas of municipalities in the state of Rio de Janeiro, Brazil between 2013 and 2017. Active procedures consisted of dragging or flagging, visual examination of vegetation and removal of ticks present on the authors’ clothing or on infested dogs. Overall, 222 ticks were collected. The altitudes at the collection sites ranged from 98 to 1220 m a.s.l. We noted a significant difference in the altitudinal distribution of *A. aureolatum* and *A. ovale* (Mann–Whitney U-test, *U* = 518.5, *P* < 0.001). The overlap of these two species occurred at altitudes of between 650 and 900 m a.s.l. The results indicated that the higher the altitude, the greater the probability for the occurrence of *A. aureolatum* and, conversely, the lower the likelihood for the occurrence of *A. ovale*. The findings of this study improve currrent knowledge on the bioecology of these tick species and have implications for studies on the epidemiology of spotted fever in Brazil.

**Graphical Abstract:**

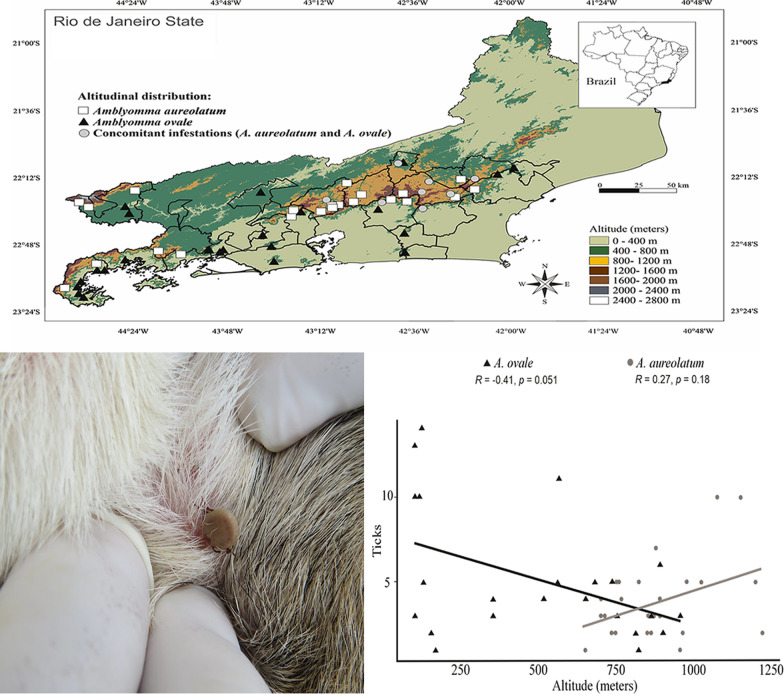

*Amblyomma aureolatum* (Pallas) and *Amblyomma ovale* Koch are widely distributed throughout the territories of Brazil [1–3]. *Amblyomma ovale* has been recorded in all Brazilian biomes, while *A. aureolatum* has recorded predominantly in the colder regions of southeastern and southern Brazil, including, high-altitude environments in the state of Rio de Janeiro [1–6]. Adults of both species feed primarily on wild carnivores, whereas immature life stages feed mainly on birds and small rodents [7–9]. In addition to wildlife hosts, both species may parasitize domestic dogs, which generally become infested when in forest environments, including the Atlantic Rain Forest, and infestations of humans have also been reported [1, 3, 7, 10]. Although the main vector of *Rickettsia rickettsii*, the causative agent of Brazilian spotted fever (BSF) in humans in Brazil is *Amblyomma sculptum* Berlese, *A. aureolatum* has been shown to transmit *R. rickettsii* to humans in the metropolitan region of São Paulo [11] and to transmit *Rangelia vitalii* to dogs [12] in southern Brazil. Furthermore, “*Canditatus* Rickettsia paranaensis” [13], a *Rickettsia parkeri*-like emerging pathogen of the New World, which is responsible for a milder spotted fever rickettsiosis in Brazil [14], has been detected in *A. ovale* [15, 16]. In addition, a single male of *A. ovale* collected from a domestic dog in Paraty City, state of Rio de Janeiro was reported as infected with *Rickettsia felis* [12].

Information on altitudinal effects on tick distribution in Brazil is scarce, with most of the records incidental and limited to an association with dogs included in studies of tick-borne diseases. In the specific case of *A. aureolatum* and *A. ovale*, the results which are available indicate that the former species prefers much higher altitudes than the latter, but also that they may occur sympatrically in some areas. This distribution was found by Sabatini et al. [17] who reported free-living specimens of *A. aureolatum* at altitudes of higher than 700 m a.s.l. and *A. ovale* at altitudes of lower than 70 m a.s.l. They also reported the occurrence of both species at an altitude of 993 m a.s.l. in a survey conducted in an Atlantic Rain Forest Reserve in the State of São Paulo. In an earlier study, Medeiros et al. [2] reported *A. aureolatum* and *A. ovale* infesting the same individual dog at lower altitudes (< 100 m a.s.l.) in the state of Santa Catarina, southern Brazil. Ogrzewalska et al. [18] collected only *A. aureolatum* from dogs at altitudes of 765–1000 m a.s.l., whereas Szabó et al. [10] reported only *A. ovale* in dogs and from the environment at 23 m a.s.l. in different areas of the Atlantic Rain Forest in the state of São Paulo, southeastern Brazil. Importantly, Barbieri et al. [3], in a specifically designed study conducted in São Paulo state, reported that the probability of finding *A. aureolatum* in municipalities situated between 101 and 700 m a.s.l. was ninefold higher than that in municipalities situated at 100 m a.s.l., or 31.5-fold higher in municipalities located above 700 m a.s.l. when compared with municipalities located 100 m a.s.l.

The aim of this short report is twofold: (i) to report new municipality records of *A. aureolatum* and *A. ovale*; and (ii) to investigate possible ecological differences in relation to altitude between *A. aureolatum* and *A. ovale* in the state of Rio de Janeiro, southeastern Brazil.

All ticks were collected in rural areas in municipalities located within the state of Rio de Janeiro between 2013 and 2017 (Fig. 1). Ticks were recovered from dogs and humans and directly from the environment (host-questing ticks), either by active or passive procedures. Active procedures consisted of dragging or flagging, visual searches on vegetation and removal of ticks from the authors and infested dogs. Passive tick surveillance consisted of veterinarians submitting ticks for examination which had been collected on dogs or on themselves and/or their clothing. Ticks infesting dogs were collected manually after physical restraint with a canvas fabric muzzle. Only nymphs and adults were collected in the present study. After collection, all ticks were placed into plastic vials containing 70% ethanol for subsequent morphological identification according to the recommendations of Dantas-Torres et al. [19].

**Fig. 1:**
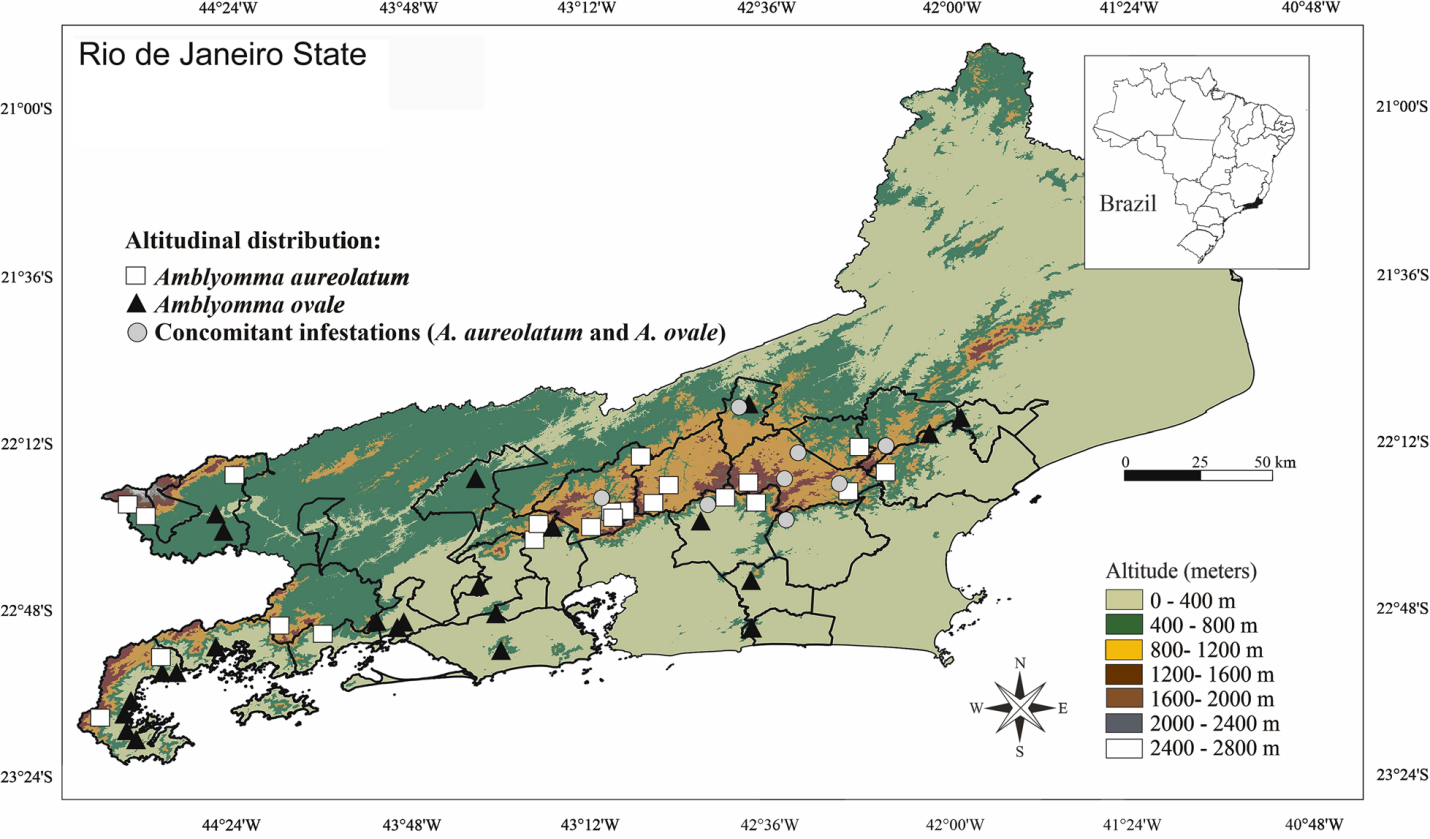
Spatial distribution of the occurrence of *Amblyomma aureolatum* and *A. ovale* in different altitudes of the state of Rio de Janeiro, southeastern Brazil

For statistical analysis, each specimen was considered to be a single tick record. The altitudinal records, recorded as meters above sea level, did not present normality by the Shapiro–Wilk test (*W* = 0.89135; *P* < 0.001). The probable differences in altitude among the municipalities of records in terms of both tick species were compared by the Mann–Whitney test, followed by the Spearman’s rank-order correlation test (Spearman’s correlation coefficient* r*_s_) [20]. All analyses were performed using R version 4.1.2 [21].

All records of both ticks were either from rural or forested areas. Ticks were collected in 26 municipalities, totaling 50 sites at different altitudes (Fig. 1). In some municipalities, more than one collection was made per site. The altitudes of the collection sites ranged from 98 (*A. ovale*) to 1220 m a.s.l. (*A. aureolatum*) (Tables 1, 2). Overall, 222 ticks were collected. *Amblyomma aureolatum* was collected at 22 (44%) sites, *A. ovale* at 23 (46%) sites and five (10%) of the sites contained both *A. aureolatum* and *A. ovale*, distributed among the following sources: 51 dogs (75.6%), 11 humans (16.2%) and six (8.2%) directly from the environmental sites (Table 1). In terms of hosts/environment, *A. aureolatum* was collected from 28 dogs, six humans and two environmental sites, and *A. ovale* was collected from 23 dogs, five humans and four environmental sites. Regarding concurrent infestations, both tick species were collected in eight sites located in six municipalities on dogs (seven individuals) and directly from the environment (two sites). There was a significant difference in terms of altitudinal distribution of *A. aureolatum* and *A. ovale* (Mann–Whitney U-test, *U* = 518.5, *P* < 0.001). It was determined that *A. aureolatum* showed a preference for higher altitudes, while *A. ovale* was recovered more frequently from sites located at low altitudes (Fig. 2). As shown in the scatter diagram (Fig. 2), a clear overlap exists in the altitudinal stratification of *A. aureolatum* and *A. ovale*, particularly at altitudes between 650 to 900 m a.s.l., based on specimens collected from seven dogs (37 ticks) and two environmental sites (nine ticks). In the case of *A. aureolatum*, there was a positive or increasing linear relationship, whereby the higher the altitude, the higher the occurrence of ticks of this species; however, Spearman’s correlation coefficient was not significant (*r*_s_ = 0.27, *P* = 0.1799). In contrast, a decreasing or negative linear relationship was observed for *A. ovale*, whereby, the higher the altitude, the lower the occurrence of that species; however, once again the Spearman's correlation coefficient was not significant (*r*_s_  = − 0.41, *P* = 0.0511).

**Table 1:** Records of altitudes at which the hard ticks *Amblyomma aureolatum* and *Amblyomma ovale* were collected in municipalities of Rio de Janeiro state from 2013 to 2017

Host	*Amblyomma aureolatum*			Altitude range (m a.s.l.)	Municipalities (total of ticks)
	AA	NN	*T*		
Dog					
28	69	10	79	650–1073	Miguel Pereira (2), Nova Friburgo (27)^a^, Petrópolis (10), Itatiaia (13), Mangaratiba (5), Paraty (3), Cachoeira de Macacu (5)^a^, Rio Claro (3), Macaé (3) Teresopólis (3)^a^, Bom Jardim (4), Sumidouro (1)^a^
Human					
6	17	0	17	764–1220	Teresópolis (5),Petrópolis (4), Paraty(2),
					Nova Friburgo (2), Mauá(2),
					Duque de Caxias (2)
Environment					
2	3		3	862–956	Trajano de Moraes (2)^a^, Silva Jardim (1)^a^
*Semi-total*	89	10	99		

Host	*Amblyomma ovale*			Altitude	Municipalities (total of ticks)
	AA	NN	*T*		
Dog					
23	86	3	89	98–890	Queimados (4), Saquarema (4), Rio de Janeiro (11), Resende (4), Itaguaí (3), Sumidouro (4)^a^, Angra dos Reis (2), Paraty (37), Cachoeiras de Macacu (5)^a^, Macaé (3), Nova Friburgo (6)^a^, Teresópolis (6)^a^
Human					
5	19	0	19	98–956	Paraty (10), Nova Iguaçu (2), Itaguaí (1),
					Rio Bonito (3), Silva Jardim (3)^a^
Environment					
4	15	0	15	560–900	Conceição de Macabu (5), Vassouras (5), Duque de Caxias (2), Trajano de Moraes (3)^a^
*Semi-total*	120	3	123		
*Total*	209	13	222		

AA, Adults; NN, nymphs; T, AA + NN

^a^Concurrent infestations

**Table 2:** Distribution of the altitudes at which *A. aureolatum* and *Amblyomma ovale* were collected in municipalities of Rio de Janeiro state from 2013 to 2017

*Amblyomma*	Altitudinal values (m a.s.l.)						
	Minimum	Maximum	Median	First quartile (Q1)	Third quartile (Q3)	Standard deviation	Coefficient of variation
*A. aureolatum*	650	1220	862	751.0	960	154.75	17.67
*A. ovale*	98	956	560	138.5	781	318.37	64.46

**Fig. 2:**
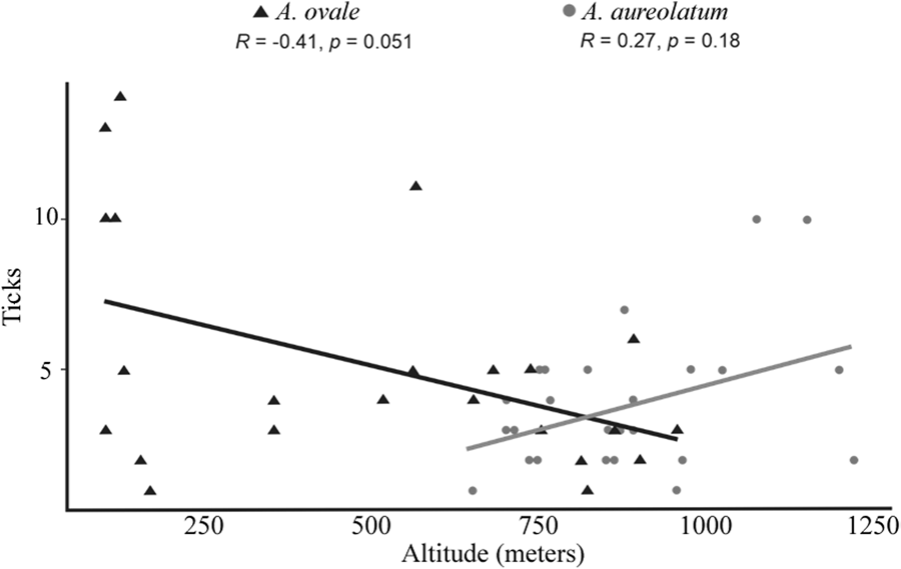
Relationship between altitudinal values (in meters above sea level) from which ticks were collected in municipalities of Rio de Janeiro State from 2013 to 2017

Based on our data, we conclude that the higher the altitude, the greater the probability for the occurrence of *A. aureolatum* and, conversely, the lower the likelihood for the occurrence of *A. ovale.* This conclusion is substantiated by data generated from an ongoing survey of ticks on dogs whose owners reside in the Environmental Protection Area of Palmares (EPAP), which is located in the municipality of Paty do Alferes, state of Rio de Janeiro, at altitudes of 840 to 1000 m a.s.l. All ticks collected on dogs were identified as *A. aureolatum* (119 adult ticks collected on 43 dogs), which one adult *A. ovale* collected from a single dog. Our findings are broadly similar to those obtained by Barbieri et al. [3] regarding altitudinal differences between the occurrence of *A. aureolatum* and *A. ovale* in the state of São Paulo. The mean altitudes of *A. aureolatum* (862 m a.s.l.) and *A. ovale* (560 m a.s.l.) observed in the present study were close to those reported by Barbieri et al. [3] (757 and 596 m a.s.l., respectively). In contrast to the data of Barbieri et al. [3] generated in São Paulo state (100–700 m a.s.l.; 23 ºS latitude), we found convincing evidence for overlapping of the two species at altitudes between 650 to 956 m a.s.l. (22 ºS). At a lower latitude (20° S), in the state of Espírito Santo, Acosta et al. [22] also reported an overlapping of *A. aureolatum* and *A. ovale* at altitudes above 700 m a.s.l.. In contrast, studies conducted in the south of Brazil, at higher latitudes (26–27 °S), the overlapping of *A. aureolatum* and *A. ovale* was observed at a far lower altitudinal range (below  100 m a.s.l.) by Medeiros et al. [2] and Barbieri et al. [23]. These contrasting ranges of tick occurrence could be explained by climate variation (winter and summer) in relation to latitude, as was discussed by Barbieri et al. [3]. At higher latitudes (e.g. southern Brazil), winters tend be more severe/colder in low-altitude regions than they are at the equivalent altitudes within regions at lower latitudes, thus favoring the establishment of *A. aureolatum*. However, in regions at lower latitudes, as was the case in the present study (22° S), winters are generally severe only at higher altitudes, with warmer summers at both low and high altitudes. In these environments, *A. aureolatum* can be expected to occur predominantly at high altitudes. Higher altitude regions (e.g. altitudes above> 900 m a.s.l.) of Rio de Janeiro state, such as Petrópolis, Teresópolis, Friburgo (Rio de Janeiro state), have an average annual temperature  < 21 °C, but can have periods with negative temperature values in winter [24]. Conversely, the altitudinal range of *A. ovale* can extend to higher altitudes (≈ 700–900 m a.s.l.) where the winter is theoretically cooler than regions found at lower than 700 m a.s.l. (less severe winters and hot summer). This observation may indicate that locations in the state of Rio de Janeiro at higher altitudes (e.g. 700–900 m a.s.l.) present favorable abiotic conditions for *A. ovale* when compared to the same altitudes in the southern region of the country, due to an increased temperature that is a consequence the lower latitude. In this context, Luz et al. [9] found both *A. ovale* and *A. aureolatum* above 700 m a.s.l., at 22° S latitude, in the Atlantic Forest of the state of Rio de Janeiro, and Szabó et al. [25] found *A. ovale* at 863 m a.s.l. in an area of Savanna, at 18°S latitude, in the state of Minas Gerais. Moreover, Acosta et al. [22] found *A. ovale* at 720 m a.s.l., at 20° S latitude. Under these circumstances, one may assume that temperature, as affected by latitude and altitude, interferes with/has an effect on the occurrence of *A. aureolatum* or *A. ovale.* This hypothesis is confirmed by the wide distribution of *A. ovale*, occurring in all Brazilian biomes [1, 3, 7, 13, 15, 25], and the more restricted occurrence of *A. aureolatum*, being concentrated in cooler environments in the south of the country and in some high-altitude regions of the southeast [2, 3, 7, 22].

Nieri-Bastos et al. [15] collected ticks from dogs in the state of Bahia, Brazil, at locations below latitude 12 ºS and at an altitude of ~ 900 m a.s.l. and reported only the occurrence of *A. ovale*. In addition, findings from field observations conducted in the Amazon biome (2 S°, state of Maranhão, Brazil), where *A. aureolatum* is absent and *A. ovale* is frequently recorded, demonstrated that < 20% of *A. aureolatum* eggs hatched when exposed to high temperatures (~ 30 °C summer) (Hermes R Luz, unpublished data). In these conditions, it is possible to speculate that *A. aureolatum* would be more sensitive to high temperatures (~ 30 °C) than *A. ovale*. Based on that presumption, and considering the predictions of the increase in the global average temperature in the coming decades (1.5–3.5 °C) [26], it may be reasonable to predict that the current distribution range of *A. aureolatum* will decrease, simultaneously accompanied by an expansion of the range of *A. ovale*. However, the data from the present study are not sufficient to support such speculations. Further experiments are necessary to clarify the effects of temperature on the biology and distribution of these ixodids in nature. In addition, it is pertinent to note that dogs whose owners live in areas of environmental protection generally move freely between high- and low-altitude areas, potentially contributing to the transport of ticks between areas at different altitudes.

The results presented herein, in combination with those reported in the literature, are of enormous importance for improving our understanding of the biology and distribution of *A. aureolatum* and *A. ovale* in Brazil, and should be considered as contributing to studies, including epidemiological investigations, of BSF. In the state of Rio de Janeiro, there were approximately 160 confirmed cases of BSF and 62 (39%) deaths between 1980 and 2014, distributed over regions of low and high altitudes [27]. In all of those regions, *A. sculptum* is the only confirmed vector of BSF to humans, although the involvement of *A. aureolatum* (high altitudes) or *A. ovale* (low altitudes) remains unclear despite the fact that those species transmit, respectively, *R. rickettsii* and *R. parkeri* [28]. Interestingly, *R. rickettsii* and *R. parkeri*, *Hepatozoon canis* [29], *Babesia vogeli* and *Rangelia vitalli* [30] have all been diagnosed in dogs having contact with forest habitats at high (mountainous) and low (valleys) altitudes in the state of Rio de Janeiro, but the association with ticks, including *A. aureolatum* and *A. ovale*, as vectors of these agents has not yet been robustly and conclusively demonstrated.

## Data Availability

The data supporting the results of this paper are included in the paper.
